# Quantity and Configuration of Available Elephant Habitat and Related Conservation Concerns in the Lower Kinabatangan Floodplain of Sabah, Malaysia

**DOI:** 10.1371/journal.pone.0044601

**Published:** 2012-10-05

**Authors:** Jason G. Estes, Nurzhafarina Othman, Sulaiman Ismail, Marc Ancrenaz, Benoit Goossens, Laurentius N. Ambu, Anna B. Estes, Peter A. Palmiotto

**Affiliations:** 1 Department of Environmental Studies, Antioch University New England, Keene, New Hampshire, United States of America; 2 Department of Environmental Conservation, University of Massachusetts, Amherst, Massachusetts, United States of America; 3 Organisms and Environment Division, School of Biosciences, Cardiff University, Cardiff, United Kingdom; 4 Danau Girang Field Centre, Sabah Wildlife Department, Kota Kinabalu, Sabah, Malaysia; 5 Hutan, Kinabatangan Orang-utan Conservation Program, Kota Kinabalu, Sabah, Malaysia; 6 Sabah Wildlife Department, Kota Kinabalu, Sabah, Malaysia; 7 North of England Zoological Society, Honorary Conservation Fellow, Chester, United Kingdom; 8 Center for Regional Environmental Studies, Dept. of Environmental Sciences, University of Virginia, Charlottesville, North Carolina, United States of America; University of Zurich, Switzerland

## Abstract

The approximately 300 (298, 95% CI: 152–581) elephants in the Lower Kinabatangan Managed Elephant Range in Sabah, Malaysian Borneo are a priority sub-population for Borneo's total elephant population (2,040, 95% CI: 1,184–3,652). Habitat loss and human-elephant conflict are recognized as the major threats to Bornean elephant survival. In the Kinabatangan region, human settlements and agricultural development for oil palm drive an intense fragmentation process. Electric fences guard against elephant crop raiding but also remove access to suitable habitat patches. We conducted expert opinion-based least-cost analyses, to model the quantity and configuration of available suitable elephant habitat in the Lower Kinabatangan, and called this the Elephant Habitat Linkage. At 184 km^2^, our estimate of available habitat is 54% smaller than the estimate used in the State's Elephant Action Plan for the Lower Kinabatangan Managed Elephant Range (400 km^2^). During high flood levels, available habitat is reduced to only 61 km^2^. As a consequence, short-term elephant densities are likely to surge during floods to 4.83 km^−2^ (95% CI: 2.46–9.41), among the highest estimated for forest-dwelling elephants in Asia or Africa. During severe floods, the configuration of remaining elephant habitat and the surge in elephant density may put two villages at elevated risk of human-elephant conflict. Lower Kinabatangan elephants are vulnerable to the natural disturbance regime of the river due to their limited dispersal options. Twenty bottlenecks less than one km wide throughout the Elephant Habitat Linkage, have the potential to further reduce access to suitable habitat. Rebuilding landscape connectivity to isolated habitat patches and to the North Kinabatangan Managed Elephant Range (less than 35 km inland) are conservation priorities that would increase the quantity of available habitat, and may work as a mechanism to allow population release, lower elephant density, reduce human-elephant conflict, and enable genetic mixing.

## Introduction

As wildlife habitats and populations become increasingly isolated, assessing the relative potential for animal movement between habitat patches, known as the permeability of the landscape [1], becomes increasingly important to understanding a landscape's functional connectivity. Landscape permeability is species specific, is linked to both habitat composition and configuration in a landscape, and is spatially and temporally complex. Spatially, managers must consider movement of target species at multiple scales. Landscape connectivity that supports intra-territorial movement necessary for wildlife to access habitat resources may not maintain movement at greater spatial scales such as the inter-territorial movements influencing metapopulation function, dispersal, and range shifts [1]. Temporal considerations are also necessary as natural disturbances can temporarily reduce permeability [1]. A lack of connectivity can leave otherwise suitable wildlife habitat unoccupied [2] resulting in discrepancies between the spatial distribution of suitable habitat and species occupancy [3].

Limiting elephant (*Elephantidae*) movement options and reducing habitat area available to them have a myriad of cascading consequences. The disruption of natural movements of elephants due to human actions such as fencing, habitat reduction, fragmentation, and transformation is a cause of locally high elephant densities [4]. High elephant densities have been cited for degradation to vegetation [5] and subsequent negative effects on other species [6]. Limited connectivity in a landscape can also leave populations vulnerable by removing their ability to shift their range in response to natural landscape processes and the effects of climate change [7]. Reduction of land available to elephants can both elevate elephant densities in remaining habitat patches and increase the human-elephant interface; two phenomenon that have been linked to increased levels of crop raiding [8]–[10].

In the early 1980s, Borneo's elephants (*Elephas maximus borneensis*) in Sabah, Malaysia were estimated to number between 500 and 2,000 [11]. The estimate was updated in 2002 to approximately 1,100 to 1,600 individuals with methods primarily based on habitat availability [12]. Recent work based on distance sampling of dung piles, estimated the total population of Bornean elephants (mostly in Sabah) at around 2,000 (published estimate: 2,040, 95% CI: 1,184–3,652), all residing in the northeastern portion of the island [13].

**Figure 1:**
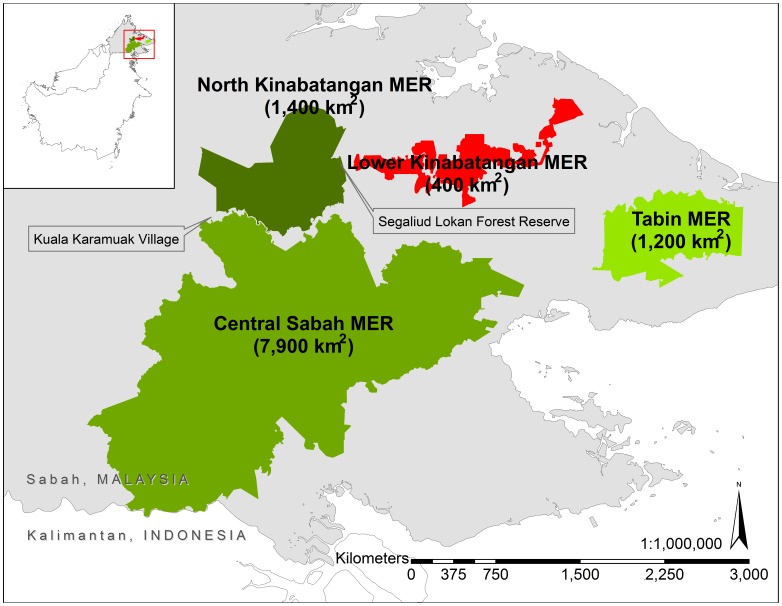
Managed Elephant Ranges of Sabah, Malaysia.

**Figure 2:**
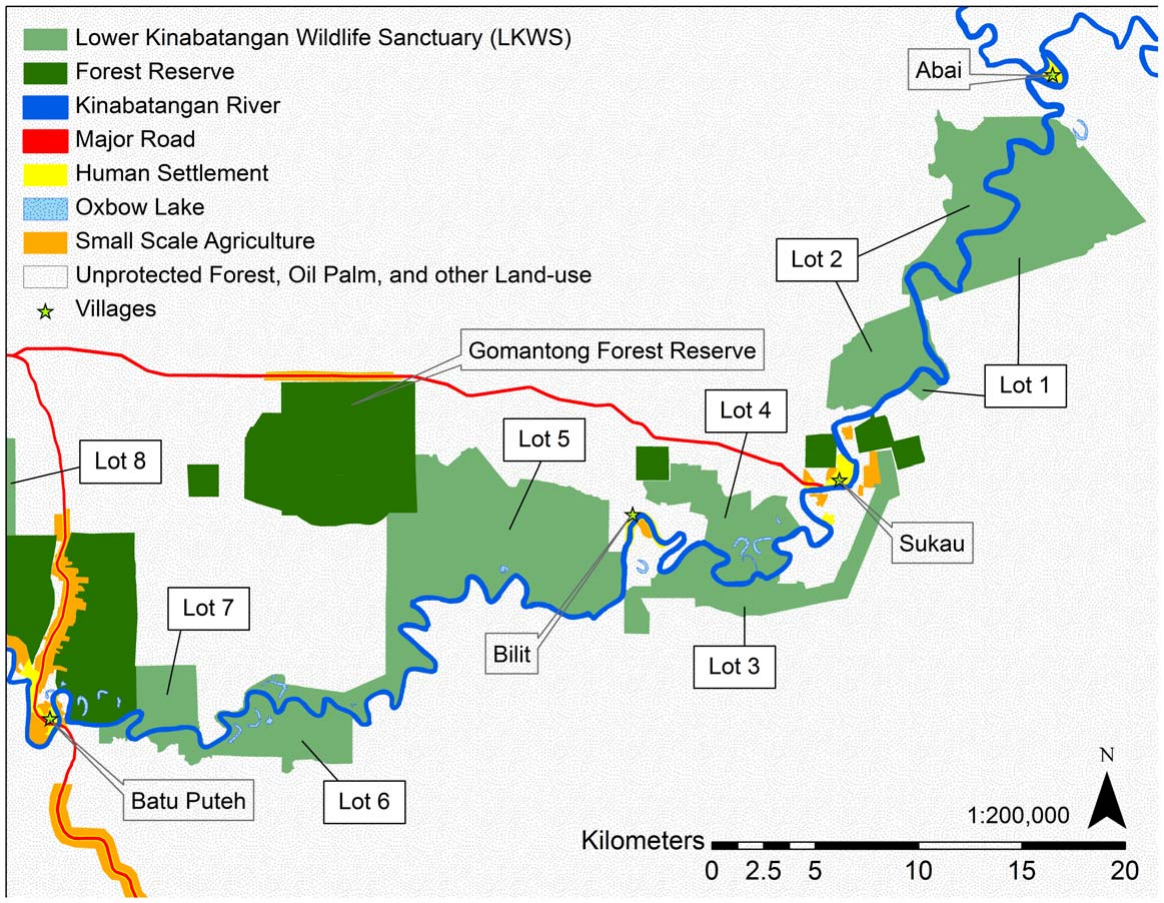
Map of the Lower Kinabatangan study area with protected forest reserves and the Lower Kinabatangan Wildlife Sanctuary.

The Sabah Wildlife Department declared four Managed Elephant Ranges: the Lower Kinabatangan (400 km^2^), North Kinabatangan (1,400 km^2^), Tabin (1,200 km^2^), and Central Sabah (7,900 km^2^), which hold over 90% of Sabah's wild elephant population in its current Elephant Action Plan (Fig. 1) [12], [14]. These ranges were based upon the current and likely future pattern and extent of forested land in the state as well as the number of elephants residing within the Ranges [12]. The Lower Kinabatangan Managed Elephant Range population is estimated at about 300 (published estimate: 298, 95% CI: 152–581) elephants [13], living within a matrix of protected and unprotected lands. Although estimates for conducting population surveys were very different, managers believe there has been a twofold increase in the ten years since the late nineties when the population was estimated at 95–115 elephants [14]. In 2005, the Lower Kinabatangan Wildlife Sanctuary successfully secured protection for 260 km^2^ of forest and connected approximately 150 km^2^ of Virgin Jungle Reserves already protected within the Lower Kinabatangan (Fig. 2). This conservation effort protects a semi-continuous corridor of natural vegetation connecting the coastal mangrove swamps with dry forests upriver in the central parts of the state. However, the overall reduction in available habitat, the disruption of elephant migration routes, and the subsequent intensification of human-elephant conflicts are all cited as conservation threats in the Lower Kinabatangan Managed Elephant Range [14].

Habitat loss is a major obstacle for elephant conservation statewide in Sabah and particularly in the Lower Kinabatangan. Habitat loss in the Lower Kinabatangan occurs directly through conversion of existing forests to other land-use such as agriculture or human settlement, while habitat isolation results from related fragmentation processes. Sabah lost approximately 40% of its forests in the 20^th^ century [15]. Over five years (1998–2003), 2,926 km^2^ of oil palm (*Elaeis guineensis*) plantation was created in Sabah, representing an average annual growth rate of 6.1% [16]. Oil palm in the Lower Kinabatangan accounts for about 28% of the total oil palm area in Sabah [17]. Plantations reduce the quantity of habitat available to elephants and are a source of conflict when elephants graze on oil palm trees. An estimated 80% of the Lower Kinabatangan floodplain has today been converted to agriculture or other human-made, non-forest land cover. Conversion of forest to agricultural land in the Lower Kinabatangan has fragmented elephant habitat and elephant populations [12]. The conversion process in the Kinabatangan is also credited with creating conflicts between elephants and people by disrupting traditional elephant migration routes, and increasing the risk of crop predation, damage to property, damage to local cemeteries, and loss of human life [12].

We suspected that available elephant habitat was overestimated by elephant management plans for the region, so we sought to identify the current quantity and configuration of available suitable habitat in the Lower Kinabatangan Managed Elephant Range. By comparing our habitat estimates with previous estimates of available habitat, we aimed to demonstrate the importance of incorporating both landscape permeability and the natural disturbance regime into elephant habitat estimates for this area. Another objective was to compare elephant density among the different habitat quantity estimates using the most recent Kinabatangan elephant population numbers available. To aid in management of this landscape we also conducted an analysis of bottlenecks in the Lower Kinabatangan. Lastly, we included discussion of what our estimates may suggest about human-elephant conflicts in the region.

## Materials and Methods

### Study area

Research was conducted in the Lower Kinabatangan floodplain of Sabah, Malaysia (approximate range of the study area is 5°18′N to 5°42′N and 117°54′E to 118°33′E). The Kinabatangan River is 560 km long, has a water catchment area of about 16,800 km^2^, and is subject to the Northeasterly Monsoonal climate. The mean annual rainfall is about 3,000 mm with the heaviest precipitation from October to March [18]. The region's matrix of habitat types include riparian forest, seasonally flooded forest, swamp forest, dry dipterocarp forest, estuary nipa palm (*Nypa fruticans*), and mangrove.

This study focused on the area between the villages of Abai and Batu Puteh (Fig. 2). Beyond Abai, elephants are restricted by vast mangrove forest. Beyond Batu Puteh, elephants are blocked from traveling upriver by a major road bordered by human settlement. The study area contains lots 1–7 (approximately 218 km^2^) of the Lower Kinabatangan Wildlife Sanctuary (LKWS) and approximately 89 km^2^ (calculation does not include mangrove forest reserves) of protected forest reserves (Fig. 2). The disturbance regime of the Lower Kinabatangan is characterized by seasonal flooding and occasional severe floods, which inundate the study area. The highest recorded flood level as of 2001 was 14 m above sea level [18]. Severe floods also occurred in 1971, 1974, 1977, 1981, 1986, 1996, and 2000. In 2000, WWF Malaysia estimated that 100 km^2^ of the Lower Kinabatangan was inundated with water for approximately 22 days. Negative effects were reported on elephants when approximately 80% of the Lower Kinabatangan Wildlife Sanctuary was flooded [18]. During the floods of 1996, inundation lasted for 35 days from the village of Sukau, about 140 km upriver, to the village of Kuala Karamuak (Fig. 1) [18]. The most recent major flood occurred in 2010.

### Available elephant habitat modeling

Landscape suitability and permeability for elephants was modeled through least-cost analysis facilitated by CorridorDesigner [19], a suite of GIS tools created for Arc GIS [20]. Four GIS layers (factors), representing land cover, linear barriers, swamps, and level of protection, were created from a combination of satellite images, scanned paper maps, field data, and local knowledge (Text S1).

A team of eight experts, all with extensive experience observing the Kinabatangan elephants, provided the weighting of the “expert opinion-based model” (Table S1). The team consisted of one professional researcher who had worked in the Kinabatangan over a decade, two graduate students studying the Kinabatangan elephants, and five local field research assistants who were employed full-time to track and study this population of elephants. The team of experts weighted each of the four factors and the suite of classes within them. Both factors and classes were given weights between 0 and 100. However, the sum of all factor scores for each expert had to sum to 100 in order to account for the relative importance of each factor. Class scores were allowed to vary independently within the weighting range.

Weighting of factors and classes occurred during an in person meeting to allow participants to ask clarifying questions throughout the process. Participants assigned weights without knowledge of other participants' weights. The experts based their opinions on their field observations of the Kinabatangan's elephants and the current body of knowledge about Bornean elephants and elephant populations elsewhere. Each participant score was given equal weight with opinions averaged for use in the “expert opinion-based model.”

In the land cover factor, the expert opinion-based model assigned high suitability to regenerating forest, also known as logged over forests, since these areas are believed to hold the highest proportion of forage for elephants. Riparian habitat along the Lower Kinabatangan River, often characterized with sandy substrate and open grassy areas, was also rated with high suitability. Human settlements were given a low suitability score since people tend to push elephants out of these areas. Similarly, oil palm plantations, although holding a great deal of forage for elephants, were rated low due to the fact that people generally protect them from elephants. Electric fences and trenches in the linear barriers factor layer, and the three swamp classifications in the swamps layer, were given low suitability scores since these have been observed to inhibit elephant passage. CorridorDesigner operates under the assumption that habitat suitability is synonymous with permeability while landscape resistance is defined as the inverse of suitability.

Factor and class weights were used to create the expert opinion-based Habitat Suitability Model (HSM). The four factors were combined with a geometric mean algorithm to reflect that one factor, such as linear barriers in the Lower Kinabatangan, can limit suitability of the pixel in a way that cannot be compensated by other factors [19]. Since an important aspect of least-cost analyses is Euclidean distance, two artificial habitat patches were placed as endpoints behind the known upriver and downriver extents of the Kinabatangan elephants' range. This was done to give the models “room to run,” so that the modeled corridor would not simply represent the shortest distance between the starting and ending habitat patches [19]. Providing source and destination points allowed us to take our point specific HSM (cost surface), and address route specific connectivity [21] with a cost-distance surface. The cost-distance surface combines cost of travel for an elephant with distance. This cost-distance grid was then used to generate a least-cost corridor model, illustrating the two dimensional configuration of the most permeable pathway between the two endpoints.

In addition to the expert opinion-based model, 21 other HSMs, cost-distance grids, and least-cost corridors were created using biologically plausible alternatives with input weights ranging within plausible bounds (Table S1). Varying the weights of model inputs provided variation in outputs. One assumption is that the modeler is able to produce much of the variation in model outputs that could be attained from various combinations of model weights falling within reasonable bounds. These alternative models were used to conduct uncertainty analysis on the final linkage design (the Lower Kinabatangan Elephant Habitat Linkage) of available suitable elephant habitat.

The expert opinion-based model and one alternative model, which identified a different pathway around the village of Sukau, were merged in order to capture the majority of variation among all models. This output was considered the most permeable suitable portion of the landscape if an elephant was traveling between the two endpoints and was referred to as the least-cost corridor.

**Figure 3:**
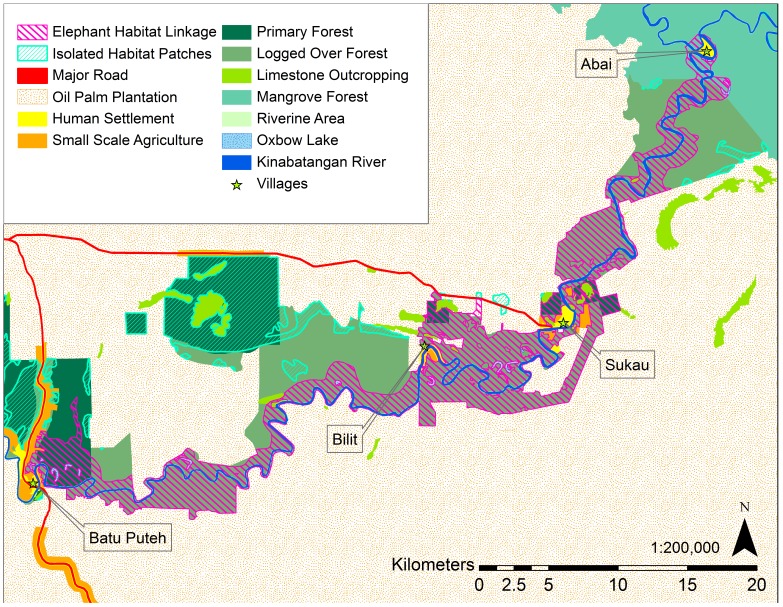
Map of the Elephant Habitat Linkage and isolated suitable elephant habitat patches.

The Elephant Habitat Linkage, representing all available suitable habitat for elephants in the Lower Kinabatangan floodplain, was created by merging the least-cost corridor with all intersecting habitat patches. Elephant habitat patches (Fig. 3) for the entire study area were determined using CorridorDesigner tools, with a 50 m moving window, a minimum patch size of 1000 m^2^, and the expert opinion-based HSM.

### Uncertainty analysis

Uncertainty analysis was conducted to assess how susceptible the least-cost corridor and the Elephant Habitat Linkage were to variations in model weighting and design. Uncertainty analysis addresses how much the model output varies in response to uncertainty in the input parameters. The model can be considered robust to uncertainty, if the range of assumptions is wide enough to be credible and the subsequent inferences are narrow enough to be useful [22], [23]. To calculate the amount of variation captured by the least-cost corridor and the Elephant Habitat Linkage, the proportion of area of each of the 21 alternative models contained within the least-cost corridor and the Elephant Habitat Linkage was calculated [22].

Both the least-cost corridor and the Elephant Habitat Linkage were robust to uncertainty in input parameters (Fig. S1). The minimum proportion of any alternative model captured by the area of either the least-cost corridor or the Elephant Habitat Linkage was 92.5%. The least-cost corridor captured an average of 97.5% of the 20 alternative models, while the Elephant Habitat Linkage captured an average of 99.3% of all alternatives. Although almost all variation among the models was captured by the Elephant Habitat Linkage, we do acknowledge that much of the Elephant Habitat Linkage is hemmed in by fenced oil palm plantations, which can explain some of this lack of sensitivity in model weighting [22]. Other modeled corridors including Beier et al. (2009), Schadt et al. (2002), and Larkin et al. (2004) also reported robustness to uncertainty in model inputs [22], [24], [25].

### Habitat quantity and elephant density analysis

Four different area calculation methods of available habitat for elephants in the Lower Kinabatangan for elephants were compared to examine the differences in habitat quantity. The first area calculation was taken from Sabah's most recent Elephant Action Plan which represented the Lower Kinabatangan Managed Elephant Range as 400 km^2^ [14]. The second area calculation, termed visually contiguous forest, consisted of all forested and riparian area, excluding mangrove forests, which would appear contiguous if looking at aerial images. The Elephant Habitat Linkage provided the third available habitat area calculation, representing both functional connectivity and habitat suitability specific to the Lower Kinabatangan's elephants. The fourth area calculation was the Elephant Habitat Linkage area minus all areas inundated with water during severe floods. The flood zone map layer was based on map created by WWF Malaysia using aerial photos of flood levels recorded in 1996 (Text S1). The area of the flood zone within the study area was calculated after digitizing the flood zone. The full area of the remaining Elephant Habitat Linkage was calculated including the area of each habitat fragment over 0.005 km^2^. The ratio of flood zone total area and Elephant Habitat Linkage area was calculated to compare the size difference between natural flooding disturbance and available suitable elephant habitat.

Elephant density was calculated for each of the available habitat area estimates by dividing the area of available habitat by the number of elephants in the Lower Kinabatangan. Since estimating elephant numbers was beyond the scope of this study, elephant density in the Kinabatangan was based on a recently conducted population survey (298, 95% CI: 152–581) based on distance sampling of dung piles [13]. We also used this estimate since it is the estimate used in Sabah's Elephant Action plan for 2012–2016 [14].

### Bottleneck analysis

Bottlenecks were defined as any portion of the Elephant Habitat Linkage centerline where the width was less than one km. This width was chosen to be consistent with successful elephant corridor width thresholds elsewhere in Asia [26]. The length that each bottleneck remained below the threshold was calculated. Each bottleneck was put into one of three categories depending on whether human-created or natural landscape features caused the bottleneck. Human features included landscape elements such as fences, human settlement, and agriculture. Natural landscape features included elements such as oxbow lakes, limestone outcroppings, and large swamps. Categories for identifying bottleneck cause included: human/human, where the bottleneck was bordered by human features on each side of the centerline, natural/natural, where natural landscape features were on each side of the centerline, and human/natural, where a combination of human and natural features caused the bottleneck.

## Results

### Modeled available elephant habitat: quantity and configuration

The Elephant Habitat Linkage (Fig. 3) covers an area of approximately 184 km^2^ and is our estimate of the current quantity and configuration of available suitable elephant habitat in the Lower Kinabatangan Managed Elephant Range. It passes close to the villages of Abai, Sukau, Bilit, and Batu Puteh. As a thin strip following the Kinabatangan River, the Elephant Habitat Linkage has an average width of only 1.3 km. At its widest, the Linkage spans 3.3 km, while the narrowest portion is 0.1 km. The widest portions include Lower Kinabatangan Wildlife Sanctuary lots 2, 6, and the combination of lots 3 and 4.

The area with visual connectivity of forests in the Lower Kinabatangan was estimated at 351 km^2^. This area is 12% smaller than the most recent documented area of the Lower Kinabatangan Managed Elephant Range. The Elephant Habitat Linkage, at 184 km^2^, is a reduction in area of 54% from the published Lower Kinabatangan Managed Elephant Range and 47% from our visual connectivity estimate.

During times of extensive flooding (as seen in 1996), approximately 423 km^2^ of the study area is inundated with water and more than 120 km^2^ of the Elephant Habitat Linkage is flooded, reducing the area available to elephants within the Elephant Habitat Linkage by 66% (62 km^2^ Elephant Habitat Linkage remaining). Inundation for much of this area lasted for 35 days [18]. Floodwaters break the Elephant Habitat Linkage into 23 fragments over 0.005 km^2^ that range in size from 31 km^2^ to 0.007 km^2^ (mean = 2.68 km^2^, SD±6.67 km^2^), representing a total area (62 km^2^) that is only 15% of the size of the Lower Kinabatangan Managed Elephant Range (Table 1).

**Table 1:** Habitat area and elephant density estimates.

*Area Estimate*	*Area (km^2^)*	*Density (elephants km^−2^)*
Lower Kinabatangan Managed Elephant Range [12]	400.00	0.75 (95% CI: 0.38–1.45)
Visually contiguous forest	350.83	0.85 (95% CI: 0.45–1.66)
Elephant Habitat Linkage	184.23	1.62 (95% CI: 0.83–3.15)
Remaining Elephant Habitat Linkage during flooding	61.75	4.83 (95% CI: 2.46–9.41)

All density estimates were based on the population estimate of 298 (95% CI: 152–581) elephants [13].

### Elephant density

Elephant density varies dramatically based on the four area calculations (Table 1). There was a 14% increase in elephant density between the documented area of the Lower Kinabatangan Managed Elephant Range and the visual connectivity approach. A 117% increase was found between the density of the Lower Kinabatangan Managed Elephant Range area and the Elephant Habitat Linkage. During flooding, the Elephant Habitat Linkage will experience a surge in elephant density by 548% compared to the Lower Kinabatangan Managed Elephant Range, and 198% compared to the Elephant Habitat Linkage.

### Bottlenecks

Twenty bottlenecks (<1.0 km width) were identified by our model throughout the Elephant Habitat Linkage. Thirty-eight percent of the Elephant Habitat Linkage's centerline was below the one km threshold. The average length for bottlenecks in the Kinabatangan was 1.9 km (max: 9.0 km, min: 0.2 km, SD±2.1). The average length between bottlenecks was 3.2 km (max: 14.0 km, min: 0.3 km, SD±4.2). Twelve bottlenecks, accounting for 44% of total bottleneck length (17.2 km), were a result of both human and natural features; four bottlenecks, representing 8% of the total bottleneck length (3.1 km), were caused by only natural features; and four bottlenecks, making up the remaining 48% (18.6 km) of bottleneck length, were caused entirely by human features.

## Discussion

### Available suitable elephant habitat

Recent landscape change, such as extensive development of oil palm plantations, has altered both the quantity and the configuration of suitable habitat for elephants in the Kinabatangan [18]. The Elephant Habitat Linkage provides a snapshot in time of our estimate of both the quantity and location of available elephant habitat in the Lower Kinabatangan.

The way in which managers classify available habitat clearly affects the quantity of habitat assumed to exist. This study's visual connectivity area estimate (351 km^2^) is 12% smaller than the documented area of the Lower Kinabatangan Managed Elephant Range. The difference likely reflects variation in the definition of available and suitable habitat but may also reflect landscape change that has occurred since the declaration of Lower Kinabatangan Managed Elephant Range. Inclusion of unreachable forest patches [27] (*Goossens et al.* unpublished data) west of the major road at Batu Puteh also contributes to the discrepancy between the Elephant Action Plan and Elephant Habitat Linkage habitat estimates.

A visual connectivity approach likely provides a more accurate estimate of available habitat compared to the Lower Kinabatangan Managed Elephant Range estimate, however it is overly simplistic. Within the Lower Kinabatangan's contiguous natural vegetation, both suitability and resistance vary due to a myriad of landscape features. Limestone outcroppings, for example, cause variation in permeability and suitability within visually connected Lower Kinabatangan forests. Limestone outcroppings often have steep slopes or cliff edges. Although these features can be small in area, they create holes of unsuitable habitat within otherwise suitable habitat and offer resistance in the landscape by acting as barriers that elephants must circumvent. Elephants are capable of navigating steep terrain when required, but have been documented in Africa to avoid gradual slopes even when forage on these slopes is optimal [28], [29]. Our results demonstrate that without considering suitability and permeability, the amount of available habitat for elephants may be greatly overestimated in the Kinabatangan. Our estimation of current available suitable elephant habitat (the Elephant Habitat Linkage) in the Lower Kinabatangan, at 184 km^2^, is 54% smaller than the Lower Kinabatangan Managed Elephant Range habitat estimate and 47% smaller than our visual connectivity estimate.

**Figure 4:**
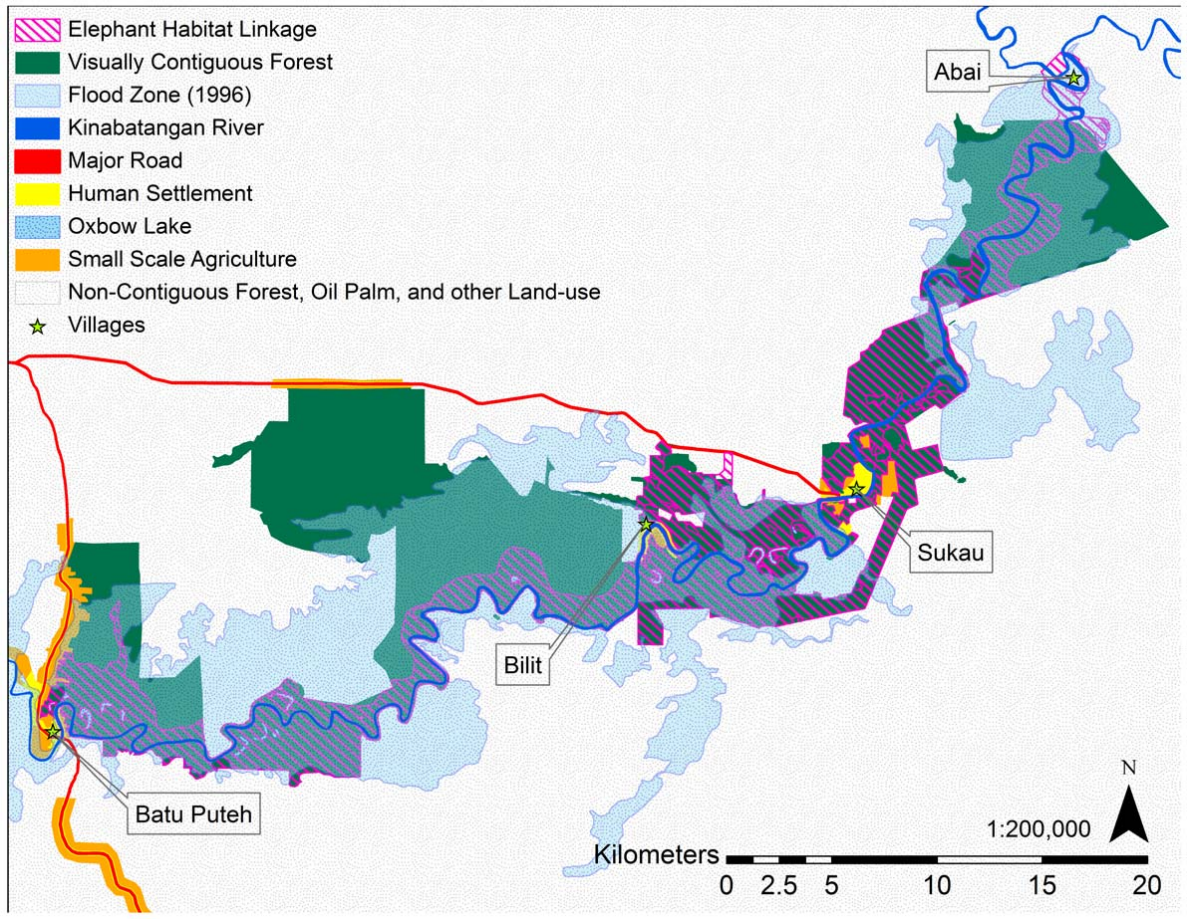
Map of visually contiguous forest, the Elephant Habitat Linkage, and the 1996 flood zone.

We took our available habitat area estimate one step further to demonstrate the importance of including natural flooding disturbance in Lower Kinabatangan elephant habitat estimates. During severe floods, the Elephant Habitat Linkage will be cut into 23 habitat fragments over the size of 0.005 km^2^ (Fig. 4) with a combined area of approximately 62 km^2^ of non-contiguous suitable habitat and an average fragment size of less than 3 km^2^. Elephants may face the risk of being temporarily trapped within these small habitat patches bordered by floodwaters, human settlements, and fenced oil palm plantations. Severe floods render 66% of the Elephant Habitat Linkage temporarily unsuitable, leaving a suitable area of only 15% the size of the Lower Kinabatangan Managed Elephant Range.

Flooding in the Kinabatangan is an important natural disturbance process that affects species, ecosystems, and landscape structure. Management areas that maintain natural disturbance regimes without the risk of negatively affecting species, ecosystems, and landscape structure probably require areas several times larger than the maximum disturbance size typical of the region [30]. The Elephant Habitat Linkage falls far short of this, with the 1996 Lower Kinabatangan flood zone (423 km^2^) more than 2.3 times its size. While we see value in managing currently unavailable habitat patches in the Managed Elephant Range for the maintenance of biodiversity and for the option to reconnect areas in the future, we stress the importance of having a more realistic awareness of the quantity of habitat available to elephants at any given time.

### Elephant density

When relatively dense populations of elephants exist, whether a result of natural population growth or growth by compression of elephants into smaller areas, undesirable changes may occur to the local habitat, vegetation, and other wildlife species [31]–[34]. Elephant density in the Kinabatangan (Table 1) surges during high flood levels to levels among the highest estimated for forest elephants anywhere in the world, comparing to 4 km^−2^ in India [35], and 3.18 km^−2^ in the Central African Republic [36]. The already high elephant density in the Kinabatangan may rise quickly if additional habitat is lost or isolated, and if managers are correct that the Lower Kinabatangan elephant population is growing at a rate that produced a twofold increase since the late 1990s [14].

There is no density of elephants that can serve as a definition of ‘overabundance’ for all areas, and a prerequisite for taking any action to manage elephant levels should include clear conservation and management objectives [37]. Elephant induced degradation of vegetation [5] which has negative effects on other species [6] have fueled debate about elephant overabundance elsewhere but has not yet been cited as a problem in the Kinabatangan. However, there is a danger of mismanaging the Lower Kinabatangan elephant population when the quantity of habitat is grossly overestimated in management plans.

Furthermore, provisional minimum home-range estimates for Bornean elephants are between 250 km^2^ to 400 km^2^ in relatively non-fragmented forests and 600 km^2^ in fragmented forests of the Kinabatangan [27]. This suggests that the Elephant Habitat Linkage may not be large enough to hold the full extent of an elephant's home range. Indeed, over 50% of GPS fixes from three collared elephants in the Kinabatangan between July 2008 and February 2009 were located in oil palm plantations (*Goossens et al.* unpublished data), possibly suggesting that elephants access a portion of their resources from behind electric fences. These resources are unavailable when plantations are effectively fenced or defended strongly by people and should be considered unreliable to support the elephant population. Conservation action is needed in the Lower Kinabatangan to increase available habitat for elephants by providing functional connectivity to existing habitat patches within the Kinabatangan (Fig. 3) and outside of the Kinabatangan to other Managed Elephant Ranges to the west.

### Bottlenecks

**Figure 5:**
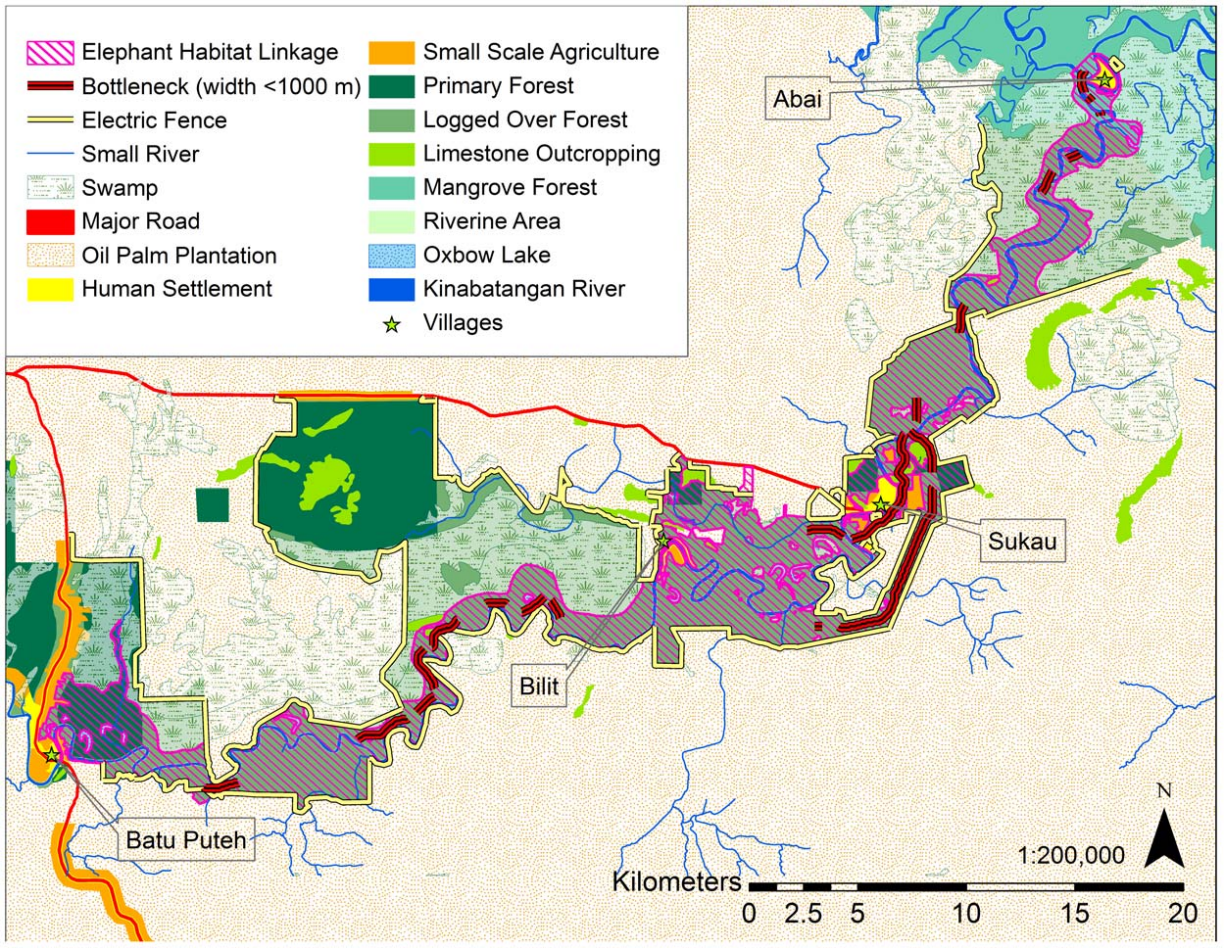
Map of electric fences, bottleneck areas, and the Elephant Habitat Linkage.

The configuration of elephant habitat in the Lower Kinabatangan jeopardizes the ability for elephants to pass throughout the Elephant Habitat Linkage. A total of 20 bottlenecks currently squeeze elephants through habitat areas where the width is narrower than 1 km. Eighty percent of all bottlenecks, and 92% of total bottleneck length, are caused at least in part by human landscape features such as oil palm plantations and human settlement. The average length of these bottlenecks is almost two km with the two longest bottlenecks (9.0 km and 6.5 km) serving as the only options for elephants to pass by Sukau Village. If elephants choose the pathway arcing farther outside Sukau through lot 3 of the Lower Kinabatangan Wildlife Sanctuary, then they would travel through more than nine km in a bottleneck approximately 0.8 km wide (Fig. 5). If they chose to travel adjacent to Sukau, they would be in a bottleneck area for about 6.5 km with width varying from 0.5 to 0.6 km or less (Fig. 5). A study in India investigated the length and width dimensions of corridors found to be used by elephants. Both bulls and family groups used passages 0.5–1 km wide and less than about five km long. It was suggested that bulls may use narrower passages, or ones with greater length to width ratios, even if these corridor dimensions were unsuitable for family groups [26]. The two bottleneck pathways around Sukau both have greater length to width ratios than seen to be effective in India, suggesting that some bottlenecks in the Kinabatangan may be nearing critical thresholds for effective use by elephants. Additionally, the average distance between bottlenecks in the Lower Kinabatangan is just over three km, which means elephants are frequently funneled into narrow passages (Fig. 5).

### Human-elephant conflict (HEC)

A combination of the quantity and configuration of available suitable habitat, high elephant densities, and bottleneck areas make the Lower Kinabatangan susceptible to high levels of HEC and put the villages of Bilit and Sukau particularly at risk. Elephants passing by Sukau must travel within one of two long, narrow bottlenecks. Bottlenecks act as funnels pinching the pathway between larger habitat patches and may cause higher incidences of conflict compared to areas outside of bottlenecks [26].

Elephants may be discouraged from using the southern passage, which takes them away from Sukau, due to its narrow northern opening caused by a combination of electric fencing and limestone outcroppings. Elephants choosing to pass closer to Sukau may frequently encounter houses and subsistence crops of local people on the south side of the Kinabatangan River.

High elephant densities resulting from population growth or habitat reduction have been associated with increased levels of HEC [8]. Additionally, the loss of elephant range [12], whether through direct habitat conversion or inaccessibility caused by isolation of habitat patches, increases the probability of contact between elephants and people by increasing the human-elephant interface [9] resulting in increased levels of crop raiding [10]. During severe floods, when the Kinabatangan experiences a surge in elephant density, the two largest Elephant Habitat Linkage fragments remaining dry are situated adjacent to the villages of Bilit and Sukau. The majority of the Kinabatangan's elephants may be trapped in these areas until floodwaters recede. If this is the case, the surge in elephant density and the proximity of the largest remaining available habitat to Sukau and Bilit may encourage HEC around these two villages.

Elephants will need to reach well-drained or higher ground when the Kinabatangan floods, which will put them in direct conflict with people. Oil palm plantation owners chose to plant on the least flood prone land and actively rid the plantations of water by digging trenches from their plantations to nearby rivers and swamps, creating bunds along the river, and installing pumping mechanisms. Elephants are left with land prone to flooding since the majority of plantations owners have installed electric fences on the plantation perimeter to exclude crop-raiding elephants. This non-random landscape change, where the comparably flood-safe land is chosen for plantations, puts elephants into conflict with people when elephants take refuge in plantations to escape floodwaters [18] and forage in areas currently planted with palm oil while other foraging areas are temporarily unavailable.

### Dispersal

Elephant dispersal is clearly limited in the Kinabatangan by a combination of electric fences, human settlements, and movement resistant natural features with low permeability such as large swamps. Gomantong Forest Reserve (Fig. 2), for example, is isolated from the Elephant Habitat Linkage by a large swamp which is bordered on each side by the electric fences of oil palm plantations (Fig. 5). Large portions of Lower Kinabatangan Wildlife Sanctuary lots 1 and 5 are modeled as unavailable due to large swamps. Combinations of barriers limiting dispersal abilities, puts the long term survival of elephants at risk by reducing their capability to respond to natural flood cycles.

Unprotected lands between sanctuaries and reserves must remain permeable in order for Lower Kinabatangan elephants to travel the extent of their current range. Predictions have doomed wildlife reserves to become islands of habitat sitting among vast inhospitable human-modified landscapes [38], [39]. However, if stakeholders in the Kinabatangan can increase the permeability of the landscape between isolated habitat patches by widening bottlenecks and establishing corridors, they may be able to reduce levels of HEC, increase elephants' abilities to respond to the natural flooding regime, and allow for genetic mixing between Sabah's elephant populations.

Managers and their partners have already established the Lower Kinabatangan Wildlife Sanctuary to provide some connectivity among other protected forests. Modern approaches of responding to overabundance of elephants in southern Africa, call for closed populations to be “released,” resulting in the decompression of populations and allowing natural processes rather than constant human intervention, to limit the effects of overabundant elephant populations [4]. If Lower Kinabatangan managers can increase available elephant habitat by reopening passages to other Managed Elephant Ranges such as North Kinabatangan Managed Elephant Range, managers may be able to avoid the expensive management of overabundant populations. The Segaliud Lokan Forest Reserve (Fig. 1) section of the North Kinabatangan Managed Elephant Range is less than 35 km inland from the main road at Batu Puteh that severs connectivity for elephants traveling westward. Sabah's 2002 Action Plan referred to the North Kinabatangan Managed Elephant Range when it stated that prospects exist for a corridor to be created which links the Lower Kinabatangan elephants with the extensive forest blocks and elephant ranges farther inland [12]. The new action plan (2012–2016) also mentions corridor creation between the Lower and Northern Kinabatangan Ranges [14]. Conservation action creating this linkage is an important step for Sabah's elephants.

### Opportunities and next steps

The methods applied here can be used for elephant conservation throughout Sabah to reassess the quantity and configuration of habitat actually available to elephants, identify areas where natural and human made barriers are working in conjunction to limit permeability or sever connectivity, locate suitable habitat patches, and generate least-cost corridors and linkage designs to identify permeable elephant movement routes with the goal of reconnecting isolated elephant habitat and elephant populations. Primary management goals should include: 1) increasing the quantity of suitable habitat in the Kinabatangan by enhancing landscape permeability and functional connectivity between the Elephant Habitat Linkage and currently isolated suitable habitat (Fig. 3), 2) widening both bottlenecks passing Sukau, and 3) reconnecting the Lower Kinabatangan elephant population with the elephants in Segaliud Lokan Forest Reserve to allow for genetic mixing and decompression of the Lower Kinabatangan elephant population.

## Supporting Information

Table S1 **Weights given to the expert opinion-based model (model 1) and the 21 alternative models (models 2–22).**
(XLSX)

Figure S1 **Percent of habitat area modeled by the expert opinion-based model (model 1) and all alternative models (models 2–22) that was captured by the Elephant Habitat Linkage area.**
(DOCX)

Text S1 **Information sources used to derive the four factor layers and flood map used in this study.**
(DOCX)
