# Supplementary material for: Quantity and Configuration of Available Elephant Habitat and Related Conservation Concerns in the Lower Kinabatangan Floodplain of Sabah, Malaysia
Source: PLoS One. 2012 Oct 5;7(10):e44601. doi: 10.1371/journal.pone.0044601 (PMC3465313; doi:10.1371/journal.pone.0044601)
Supplement: Figure S1 — Percent of habitat area modeled by the expert opinion-based model (model 1) and all alternative models (models 2–22) that was captured by the Elephant Habitat Linkage area. (DOCX) [file pone.0044601.s002.docx]

Supplementary Material

Figure S1. Percent of habitat area modeled by the expert opinion-based model (model 1) and all alternative models (models 2-22) that was captured by the Elephant Habitat Linkage area.
