# Supplementary material for: Quantity and Configuration of Available Elephant Habitat and Related Conservation Concerns in the Lower Kinabatangan Floodplain of Sabah, Malaysia
Source: PLoS One. 2012 Oct 5;7(10):e44601. doi: 10.1371/journal.pone.0044601 (PMC3465313; doi:10.1371/journal.pone.0044601)
Supplement: Text S1 — Information sources used to derive the four factor layers and flood map used in this study. (DOCX) [file pone.0044601.s003.docx]

**Supplementary material**

Text S1. Information sources used to derive the four factor layers and flood map used in this study.

*Land cover factor layer*

The land cover layer was digitized using the information sources below:

1) A parcel map originally from the Land and Surveys Department of Sabah was used to

designate boundaries of properties.

2) A soils layer (vector) created by the Bornean Biodiversity and Ecosystems Conservation (BBEC) Program in Sabah was used to identify limestone outcroppings using the Gomantong habit association in this layer.

3) A BBEC program contour map provided elevation and slope information and was one resource used to suggest limestone outcroppings. Contours were digitized into limestone outcroppings when 200 ft contours were observed without 100 ft contours. Contour lines were used to trace the shape of limestone outcroppings where identified by other sources. If other sources demonstrated a shape extending outside of the 200 ft contour, then the shape identified by the other source was followed everywhere outside the contour.

4) Mohamed et al. (2003) also indentified limestone outcroppings in the Lower Kinabatangan and reinforced the designations derived from the contour map. Mohamed et al (2003) also identified outcroppings too low in elevation to be identified by the BBEC contour map.

5) Limestone outcroppings were identified and checked during a meeting in September 2008 (pers. comm. Ancrenaz 2008).

6) Google Earth images were used as background data to distinguish land cover such as forest, oil palm plantation, and human settlement.

7) Local knowledge was employed to map recent land cover changes, to check all information, and to map features that other sources could not identify.

*Barriers factor layer*

The barriers coverage consists of linear barriers within the Kinabatangan landscape. It represents five classes including small rivers, trenches used to drain excess water from oil palm plantations, wildlife bridges built over trenches, electric fences, and all areas not considered physical linear barriers. Data for this layer was gathered from four sources:

1) Small rivers were from a map created by a project conducted under the BBEC program from 2002 to 2007.

2) Ground surveys mapped trenches and wildlife bridges using a hand held GPS unit. Trenches are dug from oil palm plantations through potential elephant habitat to the Kinabatangan River or to small rivers that subsequently drain into the Kinabatangan. In some cases wildlife bridges have been built over these trenches as an attempt to reduce the effect of trenches as barriers to wildlife movement. Elephants and other wildlife have been observed using these bridges. This study focused on trenches that drained directly into the Kinabatangan River but included other trenches found opportunistically during the study period. All oil palm trenches intersecting the Kinabatangan from Abai village to Batu Puteh village were mapped. The mouth of each trench was marked with a hand held GPS and the entire length of the trench was walked until meeting the plantation boundary or until the researchers were unable to proceed due to field conditions. If researchers were blocked from marking the location that a trench entered a plantation, the distance to the intersection point was estimated. GPS locations were taken along the trench every 100 meters. Researchers noted the presence or absence of an electric fence where the trench entered the plantation.

3) A parcel map from the Land and Surveys Department of Sabah was used to approximate the placement of electric fences to be later confirmed by field observation or local knowledge.

4) Local knowledge, and information collected from meetings with plantation managers was used to identify plantations with fences, and confirm accurate placement.

*Swamps factor layer*

The *swamps* factor was made up of four classes: sapi, kilas, mixed shrub, and not a swamp. Information for this layer came from two sources:

1) A BBEC soils layer identified sapi and kilas swamps through soil associations with these habitat types.

2) The NGO Hutan provided the spatial data for mixed shrub swamp classifications that were derived from habitat surveys.

*Level of forest protection factor layer*

The level of forest protection factor includes five classes: level V forest reserve, level 1 or VI forest reserve, Lower Kinabatangan Wildlife Sanctuary, forested area with no protection status, and non-forested areas with no protection status. Data for this layer came from three sources:

1) A BBEC map identified protected land and the level at which it was protected.

2) The Land and Surveys Department parcel map was used as another resource showing reserve boundaries.

3) Google Earth layers were used to suggest forest vs. non-forest.

4) Local knowledge was used to check the map created from the combination of sources above.

*flood zone map*

The flood zone map, based on a flood that occurred in 1996, was digitized from a scanned reference map created by WWF-Malaysia. This layer was not a factor layer used to create the Elephant Habitat Linkage.
